# ECG characteristics as indicators of the aetiology of pulseless electrical activity: a systematic review

**DOI:** 10.29045/14784726.2025.3.9.4.27

**Published:** 2025-03-01

**Authors:** Brad Gander, Samantha Laws

**Affiliations:** Critical Care Paramedic, South East Coast Ambulance Service ORCID ID: https://orcid.org/0000-0001-6836-4642; Senior Lecturer, St Georges University of London ORCID ID: https://orcid.org/0000-0002-0570-2015

**Keywords:** aetiology, ECG, pulseless electrical activity, resuscitation

## Abstract

**Introduction:**

The incidence of pulseless electrical activity (PEA) as a presenting rhythm in out-of-hospital cardiac arrest is rising in comparison with other rhythms. Prompt recognition of the cause of PEA can improve outcomes. The assessment of electrocardiogram (ECG) characteristics during resuscitation has been suggested as a source of diagnostic information for clinicians. The aim of this systematic review was to identify literature evaluating the use of ECG characteristics as indicators of the aetiology of PEA and to consider how their findings may be utilised in clinical practice.

**Methods:**

Case series, observational studies, randomised controlled trials and empirical research investigating the ECG characteristics of adult patients and reporting the aetiology of PEA were searched for via a systematic literature search of the MEDLINE, CINAHL Plus and EMBASE databases. Searches for grey literature were performed, as well as reference screening. A risk-of-bias assessment was undertaken for each included study.

**Results:**

A total of four articles were selected for final inclusion. One study reported a statistically significant correlation between the presence of wide QRS complexes and hyperkalaemia. No further associations between ECG characteristics and the aetiology of cardiac arrest were reported. Three studies were found to be at moderate risk of bias due to incomplete inclusion of patients. Studies often assessed groups of aetiologies, rather than specific causes. Consequently, this limits their application in clinical practice.

**Conclusion:**

ECG characteristics should not be used in isolation as an indicator of the aetiology of cardiac arrest in patients with PEA. The included studies often employed broad categorisations of aetiologies, limiting their ability to identify specific characteristics associated with individual causes. Future research should include analysis of specific aetiologies and the evaluation of ECG characteristics to augment other diagnostic tools.

## Introduction

Cardiac arrest is a complex condition and a significant cause of mortality and healthcare resource utilisation globally (Geri et al., 2020; Graham et al., 2015). Many initiatives aimed at improving survival from cardiac arrest have focused upon the care of patients presenting with shockable rhythms. However, the incidence of shockable presenting rhythms in out-of-hospital cardiac arrest (OHCA) has been reported to be decreasing (Keller & Halperin, 2015). Conversely, as identified by Bergström et al. (2018), the incidence of patients with pulseless electrical activity (PEA) is increasing. This retrospective database audit of 48,707 patients with OHCA revealed that the incidence of PEA as a presenting rhythm increased in every five-year period between 1990 and 2016 (p <0.0001).

Identifying the underlying cause of PEA during resuscitation is crucial for delivering appropriate interventions and improving patient outcomes (Bergum et al., 2015; Dewolf et al., 2022). However, the accurate identification of the precipitating aetiology is difficult for clinicians, as PEA is a complex clinical state with several subtypes, caused by a wide spectrum of aetiologies (Aufderheide, 2007). The evaluation of ECG characteristics during resuscitation from PEA may aid identification of the cause and required interventions. A case series and narrative review by Mehta & Brady (2012) proposed that QRS width may differ between metabolic and mechanical aetiologies. This theory has since been adapted into an algorithmic approach by Littmann et al. (2014), who advocated using the width of QRS complexes to determine the cause of PEA and then delivering subsequent treatments by using this in combination with clinical findings. This use of ECG characteristics to aid identification of the cause of OHCA in patients with PEA is included within the Joint Royal Colleges Ambulance Liaison Committee (JRCALC) guidelines for advanced life support (JRCALC, 2022). While these are theoretical models based upon small case series and expert opinion of the physiological responses seen in various conditions, the ability of ECG characteristics to identify precipitating aetiologies has not been clinically validated. Therefore, it has not been established whether these may offer accurate diagnostic insight when assessed during cardiac arrest.

A scoping search and review of the PROSPERO registry found no previous systematic reviews have been undertaken on the relationship between ECG characteristics and PEA aetiology. Van den Bempt et al. (2021) undertook a systematic literature search of pre-hospital identification of the causes of PEA; however, this only included two studies focusing upon ECG characteristics and did not critically appraise the findings. Furthermore, this review only included studies undertaken in the pre-hospital environment. Coppola et al. (2021) conducted a systematic review on the management of PEA by UK ambulance services. This reported a consensus on the lack of available evidence on the management of PEA. The authors of this review reiterated that the early recognition and appropriate treatment of reversible causes may improve survival, but concluded this is currently challenging to achieve and further evidence is needed to inform guidelines.

The aim of this systematic review is to identify and critically appraise literature evaluating the use of ECG characteristics as indicators of the aetiology of PEA, and to consider how their findings may be utilised in clinical practice.

## Methods

The Preferred Reporting Items for Systematic Reviews and Meta-Analyses (PRISMA) guidelines were used to structure this review (Page et al., 2021a).

Inclusion and exclusion criteria were determined by discussion between both authors. The criteria used are displayed within Table 1. A systematic literature search of the MEDLINE and CINAHL Plus databases was performed using the EBSCOhost platform. EMBASE was searched using the Ovid platform. These searches were performed between 22 and 31 July 2023. Search terms were determined by evaluating known articles on the subject and were piloted using selected databases. Table 2 provides the search terms used for EMBASE. Boolean operators were used to combine search terms. The full search strategies are contained within Supplementary 1.

**Table 1.** Inclusion and exclusion criteria.

Inclusion	Exclusion
Case series, observational studies, randomised controlled trials and empirical research published in peer-reviewed scientific journals with full-text availability	Single-patient case reports, neonatal or paediatric studies, animal studies
Published after 1 January 2013	Studies reporting non-standard ECG measurements
English language	Studies describing the effects of medications, electrical or mechanical cardiac support during resuscitation
Describing ECG characteristics recorded in adult patients during episodes of PEA and assessing their relationship with the aetiology of cardiac arrest	

**Table 2.** Search terms used for EMBASE.

EMBASE search terms
tachy* OR brady* OR rate OR complex* OR wide OR narrow OR QRS OR ‘p wave’ OR ‘t wave’ OR activity OR ECG OR EKG OR electrocard*
PEA OR EMD OR ‘electromechanical dissociation’ OR ‘pulseless electrical activity’
aetiolog* OR etiolog* OR cause* OR diagnos* OR condition* OR associat*
‘narrow QRS’ OR ‘narrow complex’ OR ‘wide QRS’ OR ‘wide complex’
‘ECG characteristics’ OR components
metabolic OR mechanical
hyper* OR hypo*

**Figure 1:**
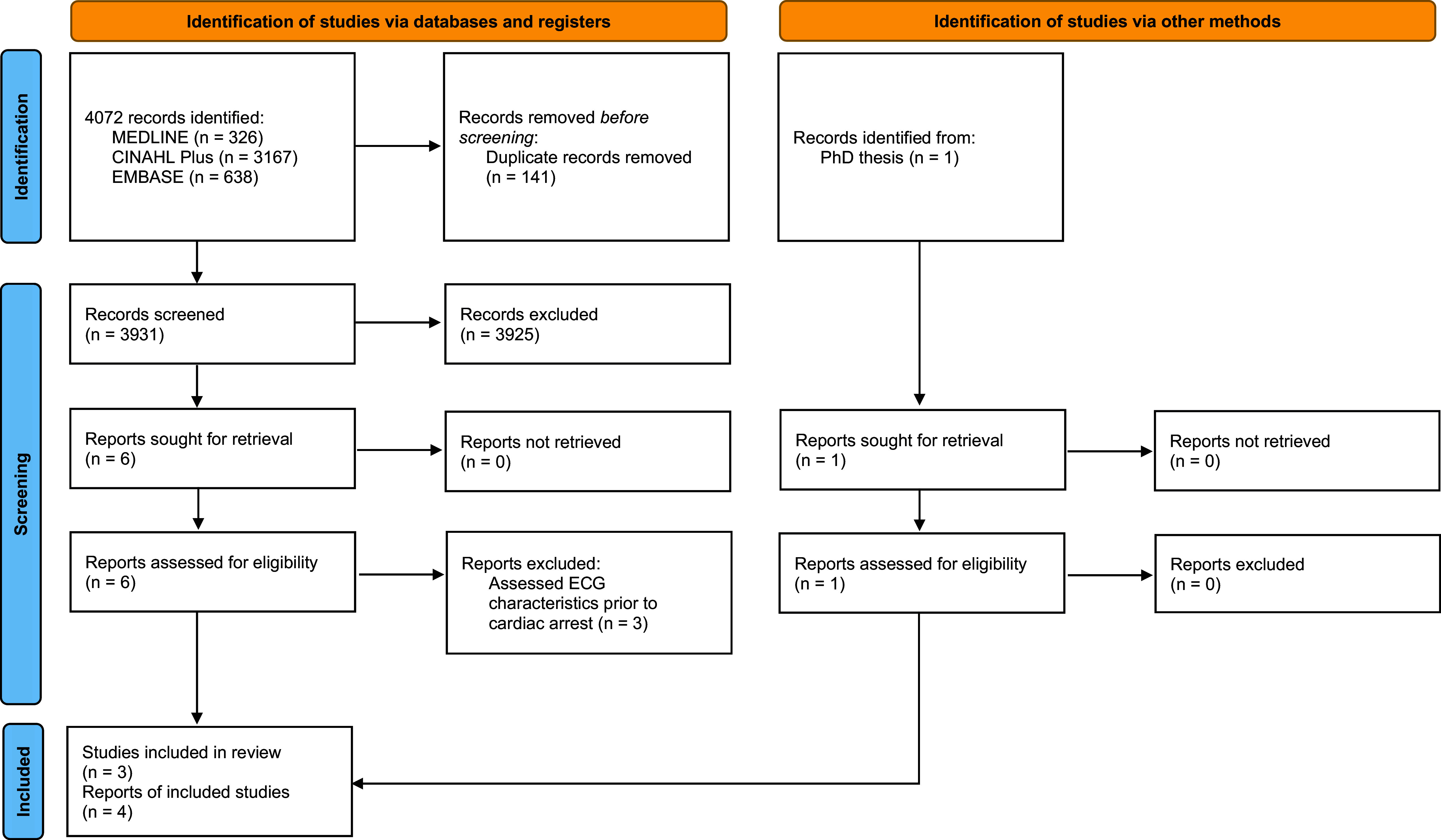
Figure 1. PRISMA flowchart.

Search results were electronically exported into Refworks™ (ProQuest, 2023) reference management software. Following the removal of duplicates, titles and abstracts were screened to identify articles suitable for inclusion. A full-text review of all selected articles was undertaken by one reviewer to determine articles for final inclusion. Instances of unclear eligibility were discussed with the second reviewer. When consensus on inclusion could not be established, a third reviewer was available to provide a final decision. The reasons for exclusion were recorded and are displayed within the PRISMA flowchart (Figure 1). Additionally, Web of Science™ (Clarivate, n.d.) was used to undertake bi-directional reference screening of eligible studies to ensure additional relevant literature was identified (Horsley et al., 2011). Academic work and publications by the authors of included studies were also screened to identify relevant grey literature.

The following data items from included studies were manually extracted by one author and recorded: sample size, ECG characteristics assessed, aetiologies assessed and results.

A risk-of-bias (RoB) assessment was performed using the JBI critical appraisal tools relevant to each study’s methodology (Supplementary 2). Overall RoB was determined by the reviewers’ overall opinion following assessment against the criteria contained within the tool.

A narrative synthesis was chosen to present the findings of this review. Meta-analysis was not performed due to the absence of intervention effect estimates and variation in assessed aetiologies and outcomes in the included studies.

## Results

Application of the electronic database search strategy yielded a total of 4072 records. MEDLINE produced 326 results, and CINAHL Plus and EMBASE produced 3167 and 638 results, respectively. After duplicate screening using RefWorks™ (ProQuest, 2023), a total of 3931 records were available for title and abstract review. Some 3925 records were removed via title and abstract screening. After obtaining the full texts of six articles, three were selected for final inclusion (Bergum et al., 2016; Kim et al., 2021; Nguyen et al., 2020). Manual reference screening identified no further articles meeting the inclusion criteria. Grey literature searching found one eligible study within a PhD thesis (Skjeflo, 2019).

A PRISMA flowchart (Page et al., 2021b) was produced to illustrate the study selection process (see Figure 1).

### Study characteristics

A total of four reports from three studies were selected for final inclusion. These comprised two published case series and one retrospective cohort study. One further case series was unpublished and contained within a PhD thesis (Skjeflo, 2019). A report of a group of patients with a specific condition without a comparator group was interpreted as a case series, as per the definition provided by Dekkers et al. (2012). The study by Nguyen et al. (2020) compared patients with bradycardic and non-bradycardic PEA and therefore was interpreted as a cohort study.

### Risk of bias in studies

The overall RoB of all included case series (n = 3) was moderate. None was able to demonstrate consecutive inclusion of patients, and two of the studies were unable to include over 50% of eligible patients due to unavailable clinical data, which gives risk to selection and reporting bias. One cohort study was included, and this was found to be at low risk of bias. The full results of RoB assessments are contained within Supplementary 2.

### Results of syntheses

The results of the individual studies are outlined in Table 3. Specific factors are discussed in more detail below.

**Table 3.** Results of individual studies.

Reference/Study type/No. of patients/Setting	ECG components assessed	Method of aetiology assessment	Aetiologies assessed	Findings	Study strengths	Study weaknesses	Notes
**Bergum et al. (2016)** Case series 51 In-hospital	P-wave Heart rate QRS duration QT interval QTc	Determined by an ‘aetiology study group’ containing anaesthesiologists, cardiologists and a pathologist after a review of patient clinical details, cardiac arrest episode and post-cardiac arrest course	Cardiac 4 Hs and 4 Ts Other (aortic rupture, sepsis, cancer) Mechanical (cardiac tamponade, pulmonary embolus, tension pneumothorax, hypovolaemia) and non-mechanical causes	No unique cause-specific pattern of ECG was identified 90% (46/51) patients had QRS >120 ms	Prospective Causes assessed using diagnostics	Single centre Small sample size	Patients from same database as Skjeflo et al. (2019)
**Skjeflo (2019)** Case series 63 In-hospital	Heart rate QRS duration (In the last 18 minutes of resuscitation prior to ROSC or death)	Determined by an ‘aetiology study group’ containing anaesthesiologists, cardiologists and a pathologist after a review of patient clinical details, cardiac arrest episode and post-cardiac arrest course	‘Cardiac’ versus non-cardiac	QRS width narrowed before ROSC and widened before death in patients with a cardiac aetiology QRS width remained unchanged before ROSC or death in non-cardiac aetiologies HR increased before ROSC in cardiac and non-cardiac aetiologies HR remained unchanged in cardiac and non-cardiac aetiologies before death	Prospective Did not use 4 Hs and 4 Ts to categorise aetiologies	Small sample size Heterogenous group of ‘other’ aetiologies Duration of resuscitation not provided No numerical data provided	PhD thesis Patients from same database as Bergum et al. (2016)
**Nguyen et al. (2020)** Retrospective cohort study 176 In-hospital	Heart rate	Review of clinical information, patient notes, resuscitation episode, death note and discharge summary by two independent investigators	All	No relationship was seen between aetiology and the development of bradycardic or non-bradycardic PEA	Used multiple hospital settings Comprehensive review of causes of arrest	Used clinician interpretation of the cause rather than diagnostic findings	
**Kim et al. (2021)** Retrospective case series 617 In-hospital	QRS duration	Laboratory test results (hyperkalaemia defined as a serum potassium level >5.5 mmol/L)	Hyperkalaemia	Narrow QRS mean potassium: 4.6 mmol/L Wide QRS mean potassium: 5.4 mmol/L 49.6% (n = 55/111) in the wide QRS group had hyperkalaemia 26.7% (n = 135/506) in the narrow QRS group had hyperkalaemia	Adjusted for confounders Large sample size	Single centre Retrospective No patient outcomes reported	

ROSC = return of spontaneous circulation, HR = heart rate, PEA = pulseless electrical activity

#### Heart rate

Three studies investigated the relationship between the heart rate (HR) and aetiology of PEA. Bergum et al. (2016) undertook a single-centre, prospective study, with the aim of evaluating the relationship between the cause of cardiac arrest and the presenting ECG characteristics in adult patients with PEA. The presumed aetiology of cardiac arrest was established via the evaluation of pre-arrest clinical information, the cardiac arrest episode and post-arrest course by a group of anaesthesiologists, cardiologists and a pathologist. Out of 144 patients who received cardiopulmonary resuscitation due to PEA, defibrillator data and a cause of arrest was available in 51 patients. Patients were divided into six groups determined by their HR and QRS width (narrow-slow, normal, narrow-fast, wide-slow, wide, wide-fast). Aetiologies were grouped into ‘cardiac’ causes or those included with the traditional ‘4 Hs and 4 Ts’ – hypoxia, hypovolaemia, hypo-/hyperkalaemia/metabolic, hypo-/hyperthermia, thrombosis (coronary or pulmonary), tension pneumothorax, tamponade (cardiac), toxins (Soar et al., 2021). When presented graphically, a wide distribution of causes across all ECG characteristic groups was seen, with no ECG patterns uniquely associated with either group of aetiologies. A further graphical evaluation of ‘mechanical’ and ‘non-mechanical’ causes also revealed no clear correlation with any ECG group. No differences were seen between aetiologies and the rate of QRS complexes during PEA. A median HR of 57 bpm (interquartile range (IQR) 41–66 bpm) was seen in patients with a ‘cardiac’ aetiology (myocardial infarction, cardiac tamponade and heart failure). In patients with an aetiology listed within the traditional 4 Hs and 4 Ts, the median HR was 48 bpm (IQR 35–65 bpm). When individual aetiologies were assessed, patients with PEA due to hypovolaemia had a median HR of 55 bpm (IQR 44–68 bpm), 46 bpm (35–60 bpm) in hypoxia and 59 bpm (52–78 bpm) in cases of myocardial infarction; however, no further analysis was performed to determine the statistical significance of these differences.

Conversely, Nguyen et al. (2020) examined the relationship between respiratory arrest and the development of bradycardic PEA, defined as an HR less than 60 bpm. This study included 176 patients, with 66 (37.5%) experiencing bradycardic PEA and 110 (62.5%) having non-bradycardic PEA. This distribution of patients is contrary to the findings of Bergum et al. (2016), which were that the majority of patients included within their study had a bradycardic HR at the onset of PEA. The cardiac arrest aetiology was determined by two independent investigators who reviewed patient notes, discharge summaries and clinical data. A total of 36.4% of patients with bradycardic PEA had a prior respiratory arrest, compared to 27.3% of those with non-bradycardic PEA (p = 0.24). This led to the conclusion that there was no association between preceding respiratory arrest and bradycardic PEA arrest. When assessing for other causes, the authors found that patients with bradycardic PEA were more likely to have an arrest caused by myocardial infarction, cardiac tamponade, electrolyte abnormalities and pulmonary embolism. Acidaemia, shock and unknown aetiologies were more prevalent in patients with non-bradycardic PEA. Due to small sample sizes, no analysis of the statistical significance of these findings was undertaken.

Finally, Skjeflo (2019) utilised the same database as Bergum et al. (2016) and included 63 patients with a diagnosed cause of cardiac arrest. This study investigated the dynamic changes of ECG characteristics during resuscitation and their relationship with the aetiology of cardiac arrest. The study categorised causes as cardiac or non-cardiac and divided them based on outcomes (return of spontaneous circulation (ROSC) or no ROSC). ECG measurements were then taken from the last 18 minutes of resuscitation before ROSC or death (no ROSC). Patients with both cardiac and non-cardiac aetiologies displayed an increase in HR prior to ROSC and no change in those without ROSC. These findings were displayed via graphs and no additional statistical analysis was provided for reference, thus limiting analysis of the differences between each group.

#### QRS width

Three studies evaluated the QRS width in PEA and its relationship with the aetiology of cardiac arrest. Bergum et al. (2016) reported that no relationship was seen between QRS duration and the aetiology within their study. In 90% (46/51) of cases with available defibrillator data there was a ‘wide’ QRS (>120 ms), and in only 6% (3/51) was there a ‘normal’ ECG pattern (QRS duration <120 ms and HR 60–100 bpm). Additionally, evaluations of the ECG characteristics of several individual aetiologies revealed that patients with myocardial infarction, cardiac tamponade, heart failure, hypoxia, hypovolaemia and pulmonary embolus all had clinically ‘wide’ median QRS widths ranging between 165–240 ms.

Skjeflo (2019) reported noticeable differences in changes to the QRS width during resuscitation between patients with cardiac and non-cardiac aetiologies. Patients with cardiac aetiology had wider QRS width, which narrowed prior to ROSC being obtained, while patients with no ROSC displayed a continually increasing QRS width during resuscitation. Patients with non-cardiac aetiologies showed little change in QRS width before ROSC or no ROSC. As previously discussed, no numerical data was provided within the results of this study, and findings were displayed within graphs.

Kim et al. (2021) focused on the relationship between QRS width and the presence of hyperkalaemia in patients with PEA. Among the 617 PEA episodes with ECG records and serum potassium measurements available, 111 had a wide QRS (>120 ms) and 506 had a narrow QRS (<120 ms). The authors found those with a narrow QRS had a mean serum potassium of 4.6 mmol/L compared to 5.4 mmol/L in those with a wide QRS (IQR 4.4–6.7 versus 4.0–5.6, p <0.0001). Patients with a wide QRS had a greater incidence of hyperkalaemia (serum potassium >5.5 mmol/L) compared to those without (26.7% versus 49.6%, p = 0.001). In the 149 patients presenting with OHCA, 108 (72.5%) presented with a narrow QRS, and 41 (27.2%) with a wide QRS. Patients with a wide QRS were more likely to be older (68.0 versus 64.0, p = 0.047), male (67.6% versus 57.3%, p = 0.047) and to have a history of diabetes (43.5% versus 31.6%, p = 0.017). After adjusting for confounding variables, wide QRS PEA was associated with hyperkalaemia with an odds ratio (OR) of 2.86 (95% CI 1.80–4.53, p <0.001). These findings were repeated in patients with chronic kidney disease (OR = 4.56, 95% CI 1.31–15.82, p = 0.017) and without (OR = 2.79, 95% CI 1.63–4.77, p < 0.001).

#### Other ECG components

Only one study investigated additional ECG components. Bergum et al. (2016) found no differences in the incidence of P-waves between aetiologies. Patients with myocardial infarction (n = 13) and heart failure (n = 5) had the longest median QT (518 ms and 595 ms) and QTc intervals (504 ms and 504 ms). Patients with hypoxia and thrombus / pulmonary embolism had the shortest median QT intervals (479 ms and 494 ms). The QTc intervals were shortest in patients with cardiac tamponade (446 ms) and thrombus / pulmonary embolism (426 ms). No statistical analysis to determine the significance of the difference between the ECG characteristics of each group was undertaken.

## Discussion

Previously proposed theoretical models of PEA have suggested ECG characteristics will reflect the pathophysiological responses to certain aetiologies seen in patients prior to cardiac arrest. For example, Mehta & Brady (2012), Littmann et al. (2014) and Jentzer et al. (2016) suggested patients with a ‘mechanical’ aetiology, such as tension pneumothorax, cardiac tamponade or pulmonary embolus, would have narrow complex, tachycardic rhythms on initial presentation. Conversely, wide complex PEA has been proposed to occur due to metabolic abnormalities (Littmann et al., 2014; Mehta & Brady, 2012). These concepts are not fully supported by the literature identified within this review, and therefore should not be used as a basis for determining resuscitation strategy in isolation. Within Bergum et al.’s study, just two out of 18 patients with a ‘mechanical’ aetiology were tachycardic, both with a wide QRS, and 10 were bradycardic. Furthermore, 90% (n = 46/51) of patients had a wide complex QRS irrespective of cause. Skjeflo (2019) also found that both ‘cardiac’ and ‘non-cardiac’ causes, defined as all other aetiologies except myocardial infarction or cardiac tamponade caused by myocardial infarction, produced clinically wide QRS complexes. A similar pattern of change in patients with in-hospital cardiac arrest was observed with the work of Attin et al. (2015), who described QRS prolongation in the last hour before cardiac arrest in all subjects within their study. This may therefore provide further evidence that the reasons for QRS prolongation in PEA are multi-faceted and cannot be attributed to isolated pathologies.

A retrospective study by Shan et al. (2022) found that patients with a respiratory aetiology frequently displayed an abrupt decrease in HR prior to cardiac arrest and suggested that this may be a cause of PEA. In these circumstances, the development of PEA may be explained by falling cardiac output secondary to bradycardia caused by myocardial ischaemia due to systemic hypoxia. However, Nguyen et al. (2020) reported a statistically non-significant difference between the incidence of bradycardic PEA (<60 bpm) and non-bradycardic PEA in patients with prior respiratory dysfunction, and found bradycardic PEA was not specific to respiratory causes alone. Attin et al. (2015) reported the development of bradycardic PEA in 82% (n = 32/39) of patients with a history of cardiac disease, and postulated that this occurs due to an imbalance between the parasympathetic and sympathetic nervous systems. Unfortunately, no aetiology assessment was performed in these subjects to provide evidence to support this theory. Further to this, Holmstrom et al. (2022) found bradyarrhythmia to be the second most dominant cause of PEA cardiac arrest, with 80% of these caused by a conduction block.

As argued by Tripepi et al. (2010), the risk of selection bias is of paramount importance in aetiological research, as it may limit the external validity of results. Three of the four studies within this review were determined to be at a moderate risk of bias due to a lack of consecutive inclusion of participants. Bergum et al. (2016), Skjeflo (2019) and Kim et al. (2021) all reported difficulty in matching ECG records with patient data, or ECG records of insufficient quality to allow for analysis. Improvements to defibrillator analysis software and the routine inclusion of defibrillation files into resuscitation registries has been suggested as a solution to this issue (Nordseth et al., 2024).

The utility of ECG characteristics in identifying patients with metabolic disturbances was only investigated in one study. Kim et al. (2021) reported a statistically significant association between wide QRS complexes and hyperkalaemia. While this may appear to support the theory that patients with wide QRS complexes may have underlying metabolic abnormalities, there are several factors inherent to the retrospective nature of this study that provide important limitations to the clinical application of these findings. As proposed by Martin et al. (1989), serum potassium may increase during cardiac arrest due to poor tissue perfusion and contribute to the widening of QRS complexes in PEA, rather than being the primary cause. Furthermore, Vallentin et al. (2022) suggested that calcium chloride may be harmful when administration is guided by ECG changes indicative of hyperkalaemia. Within their analysis, ROSC was achieved in 20% (n = 9) of patients receiving calcium chloride and in 39% (n = 23) of those treated with a placebo. Just 2.2% (n = 1) survived to 30 days in the calcium group compared to 13.6% (n = 8) receiving a placebo. This study indicates that calcium chloride should not be initiated based on ECG characteristics in isolation. This is furthermore confirmed by the findings of this review, where wide complex PEA was not demonstrated to occur due to metabolic disturbances alone.

The assessment of dynamic changes to ECG components throughout resuscitation may have the potential to yield diagnostic information and aid assessment of the response to resuscitation. Skjeflo (2019) demonstrated that the QRS width of patients with cardiac aetiologies was wider than those with non-cardiac aetiologies when recorded 18 minutes before ROSC or death. Interestingly, the QRS width then narrowed prior to ROSC in patients with cardiac aetiologies, while it remained unchanged in those with non-cardiac aetiologies. The underlying reasons for these ECG changes remain unclear. Outside the context of cardiac arrest, prolonged QRS duration has been linked to cardiovascular disease or structural cardiac issues (Chen et al., 2021). Experimental studies have also shown that myocardial ischaemia can directly affect cardiac depolarisation and conduction velocity (Almer et al., 2016; Good et al., 2021). This may explain the differences observed between cardiac and non-cardiac aetiologies, where in the latter, cardiac electrophysiology is preserved due to the absence of direct insult to the myocardium. Based on these findings, in patients with suspected cardiac aetiologies, narrowing of the QRS width during resuscitation may indicate improving myocardial perfusion. Conversely, widening QRS complexes may suggest an inadequate response to treatment and the need to consider alternative strategies.

### Limitations

This review has several limitations. Only English-language literature was included, potentially excluding relevant studies published in other languages. Additionally, subject matter experts were not consulted during the review process, which could have identified additional relevant literature. Studies published before 1 January 2013 were excluded to obtain results that reflect contemporary resuscitation practice and diagnostics. When making this decision, the references of articles proposing the use of ECG characteristics as indicators of aetiology in PEA were assessed and no relevant clinical studies were identified. Nonetheless, this date limit may still have excluded additional studies supporting the use of ECG characteristics for this purpose.

One limitation of research assessing the aetiologies of PEA lies in the respective methods of determining the correct aetiology and how they are categorised. Bergum et al. (2016) and Skjeflo (2019) grouped causes based on broad descriptions of the mechanism, such as ‘cardiac’ or ‘non-cardiac’, or ‘mechanical’ and ‘non-mechanical’, rather than specific causes. This approach limits the usefulness of ECG characteristics as indicators of specific pathologies that may require different interventions. For example, cardiac tamponade and myocardial infarction were both included within the ‘cardiac’ group of aetiologies, despite requiring distinctly different treatments. While these studies only included cases with presumed reliable diagnoses, the methods used to determine the aetiology in cases that resulted in death may lack accuracy and objectivity, as they were based on clinical opinion rather than autopsy findings. Moreover, Holmstrom et al. (2022) reported that 65% of PEA cases had an undetermined trigger in a retrospective analysis of the medical records of 97 survivors. Therefore, the true diagnostic accuracy of both studies may not be fully known.

Additionally, there is inconsistency among the included studies on the timing of assessment of ECG components. Therefore, the ECG measurements obtained may have been influenced by treatments or physiological deterioration during resuscitation and may not represent the initial ECG components at the onset of cardiac arrest.

## Conclusion

The current available evidence does not provide clear support for using ECG characteristics in isolation as reliable indicators of the underlying causes of PEA cardiac arrest. The reported values for certain subtypes of aetiology do not match the theoretical models proposed within the literature. Additionally, the methods used to determine aetiologies may lack accuracy and consistency, due to reliance on clinical opinion rather than pathological findings at autopsy.

Future research should utilise data from pre-hospital services and witnessed cardiac arrests in order to provide a more accurate understanding of the ECG characteristics in the early stages of PEA OHCA. This should include combined defibrillator or ECG data and patient clinical data. Studies focusing on monitored patients who deteriorate into PEA following a confirmed diagnosis, rather than undifferentiated cases, may enhance our understanding of the ECG characteristics associated with specific pathologies and how these manifest in early cardiac arrest. Finally, when available, autopsy data should be incorporated to improve the evaluation of aetiologies in patients who do not survive resuscitation.

## Acknowledgements

I would like to acknowledge the valuable feedback and academic support provided by Mary Halter throughout this project.

## Author contributions

This systematic review was originally undertaken and submitted as part of a Masters in Prehospital Critical Care. BG developed the aim and objectives, undertook the literature searches, data extraction and RoB assessments and drafted the manuscript. SL provided continuous supervision and secondary review throughout all areas. Both BG and SL contributed to the final version of the manuscript. BG acts as the guarantor for this article.

## Conflict of interest

None declared.

## Ethics

Ethics approval was not necessary as no primary research was undertaken and all included studies received their own respective ethics approval.

## Funding

None.

## Registration and protocol

This systematic review was originally undertaken and submitted as part of a Masters in Prehospital Critical Care; therefore it was not registered prior to commencement. A protocol was created as part of this work and is available upon request. No alterations were made to the design of the review.
